# Epidemiology of paediatric injuries in Nepal: evidence from emergency department injury surveillance

**DOI:** 10.1136/archdischild-2020-321198

**Published:** 2021-08-30

**Authors:** Dan Magnus, Santosh Bhatta, Julie Mytton, Elisha Joshi, Sumiksha Bhatta, Sunil Manandhar, Sunil Joshi

**Affiliations:** 1 Centre for Academic Child Health, University of Bristol, Bristol, UK; 2 Centre for Academic Child Health, University of the West of England, Bristol, UK; 3 Nepal Injury Research Centre, Kathmandu Medical College, Kathmandu, Nepal; 4 Mother and Infant Research Activities, Kathmandu, Nepal

**Keywords:** health services research, epidemiology, data collection

## Abstract

**Background:**

Globally, injuries cause >5 million deaths annually and children and young people are particularly vulnerable. Injuries are the leading cause of death in people aged 5–24 years and a leading cause of disability. In most low-income and middle-income countries where the majority of global child injury burden occurs, systems for routinely collecting injury data are limited.

**Methods:**

A new model of injury surveillance for use in emergency departments in Nepal was designed and piloted. Data from patients presenting with injuries were collected prospectively over 12 months and used to describe the epidemiology of paediatric injury presentations.

**Results:**

The total number of children <18 years of age presenting with injury was 2696, representing 27% of all patients presenting with injuries enrolled. Most injuries in children presenting to the emergency departments in this study were unintentional and over half of children were <10 years of age. Falls, animal bites/stings and road traffic injuries accounted for nearly 75% of all injuries with poisonings, burns and drownings presenting proportionately less often. Over half of injuries were cuts, bites and open wounds. In-hospital child mortality from injury was 1%.

**Conclusion:**

Injuries affecting children in Nepal represent a significant burden. The data on injuries observed from falls, road traffic injuries and injuries related to animals suggest potential areas for injury prevention. This is the biggest prospective injury surveillance study in Nepal in recent years and supports the case for using injury surveillance to monitor child morbidity and mortality through improved data.

**What is already known on this topic?**

- Injuries are a serious global problem for children and young people and are the leading cause of death in people aged 5–24 years globally.
- The injury burden in children in Nepal is high.
- Systems for injury surveillance in children are particularly scarce in low-income and middle-income countries

**What this study adds?**

- In children attending emergency departments in this study, the most common injuries were unintentional—specifically falls, animal bites and stings and road traffic injuries.
- The group of children most susceptible to injuries in this part of Nepal were male children under the age of 10 years.
- Establishing injury surveillance for children in emergency departments is possible and can yield important data to inform injury prevention programmes and policies.

## Background

Globally, injuries cause >5 million deaths annually—a similar number to those from HIV/AIDS, tuberculosis and malaria combined.^1^ Children and young people are a particularly vulnerable group and injuries are the leading cause of death in people aged 5–24 years globally,^1 2^ and of disability for people aged 5–44 years.^3^

The majority of global child mortality and morbidity from injuries occurs in low-income and middle-income countries (LMICs).^4–6^ Paediatric trauma has been highlighted as a global health priority,^7^ yet challenges exist to identify injured children in LMICs and conflict settings,^8^ resulting in calls for better data and for injury surveillance.^9^

Reliable estimates of injury burden in Nepal are limited.^10^ In 2017, injuries were estimated to account for nearly 10% of all deaths in Nepal, with transport injuries, falls, drowning, animal related, burn, self-harm and interpersonal violence as the leading causes.^11^ Children living in Nepal face significant health challenges, with an under-5 mortality of 39 per 1000 live births.^12^

The quality of data relating to childhood and adult injury is inversely correlated to where the greatest problems exist. Existing data on trauma in most LMICs remain poor, with LMIC injury data constituting around 1% of all data. The use of injury surveillance systems has been proposed as a way to improve this.^13^ The collection and use of data on risk factors, incidence, severity, outcomes and costs can assist in identifying populations at risk, implementing and evaluating prevention programmes and formulating and evaluating policy.^14^ This study was designed to inform injury prevention activities and policy development and is focused on surveillance and data collection tools to describe the epidemiology of childhood injury.

## Methods

Objectives of this mixed methods study were (i) to design an injury surveillance tool and data collection process; (ii) prospective collection of data on injuries presenting to two hospital emergency departments over 12 months; (iii) process evaluation to explore the barriers and facilitators to sustainability (reported separately). The published study protocol^15^ describes data collector recruitment and training, data tool testing and quality assurance. This paper reports injury cases presenting in children under the age of 18 years. The data on adult patients are reported separately.

The Makwanpur district of Nepal has an estimated population of 420 477, including 174 417 children aged 0–17 years inclusive (50.2% male).^16^ Most of the population live in rural areas (83%). Both study hospitals are in Hetauda, a submetropolitan city approximately 120 km southeast of Kathmandu. Both are secondary care hospitals and have the facilities to provide treatment for major and minor trauma.^17^ Most local injury cases attend these hospitals because of the long distances to other tertiary care hospitals and poor transportation systems. Hetauda hospital is a government-funded district hospital with 110 beds serving about 300 emergency and outpatient attendances per day. There are 19 doctors and 47 nursing and paramedic staff. Chure Hill Hospital is a private hospital with 25 beds serving about 60 emergency and outpatient attendances per day. There are 12 doctors and 65 nursing and paramedic staff (source: verbal inquiry with the hospital management authority).

For inclusion in the surveillance study, patients met the following criteria: presenting to either of the two study hospitals, with a new injury, of any cause, within 7 days of the injury event. Exclusion criteria were: repeated attendance in the same department for the same injury; previous attendance in other study site for the same injury; or injury sustained >7 days prior to presentation. Data were collected from patients presenting with injuries between 1 April 2019 and 24 March 2020 inclusive (data collection ended 1 week earlier than scheduled because of the COVID-19 pandemic). A standardised data collection form was developed from existing tools^14 18 19^ and adapted for the Nepal context. Once urgent clinical care had been given, data collectors approached the patient (or carer, where necessary and appropriate) for consent to participate. Verbal consent was recorded on tablet computers together with anonymised patient data on sociodemographics, date of injury, mechanism of injury, clinical presentation, diagnosis, severity of injury and disposition. Data collectors were trained how to categorise the diagnosis based on information collected from patients/carers, thereby reducing the risk of misclassification. The level of injury severity was agreed by the data collector in conjunction with the clinical staff and was classified depending on the level of skilled emergency care required. Data were entered electronically using Research Electronic Data Capture software,^20 21^ where they were encrypted and uploaded to a secure online database. The final non-identifiable dataset was exported for analysis using SPSS.^22^

For data analysis, frequency data were explored by age, sex, ethnic group, setting of injury event, type and mechanism of injury and disposition. Rates of injuries by age and sex were calculated using Makwanpur population estimates from the 2011 census. Associations between injury severity, sex and age group were explored using χ^2^ analysis, and two age groups (<15 years and 15–17 years) to compare injury severity in younger children with young adults.

## Results

**Figure 1:**
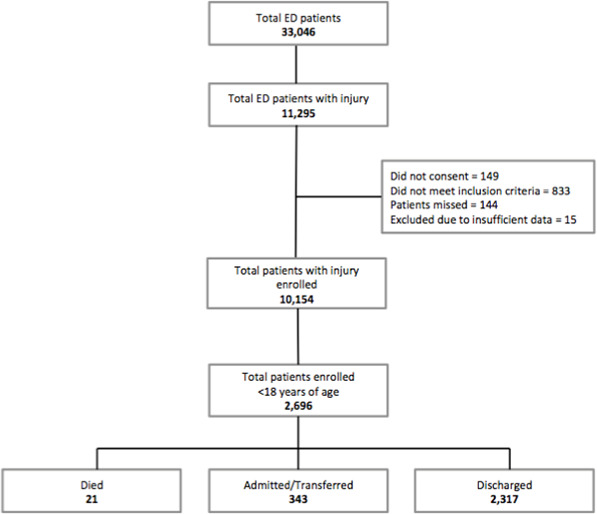
Patients with injuries attending emergency departments in Makwanpur district, Nepal, April 2019–March 2020.

The total number of patients that presented to the emergency departments during the study period was 33 046, of which 10 154 (30.7%) were patients of any age with injuries who were eligible for inclusion and consented. For the nested study reported here relating to children with injuries, 2696 (26.6%) were patients under the age of 18 years, with 2274 (84.3%) presenting to Hetauda hospital and 422 (15.7%) to Chure Hill hospital (figure 1).

Most children presenting with injuries to the emergency departments were male (66%) and <10 years of age (56.3%). Sociodemographic and injury characteristics are shown in table 1.

**Table 1:** Sociodemographic profile and injury characteristics among children attending emergency of hospitals in Makwanpur district, Nepal, April 2019–March 2020 (N=2696)

Patient/Injury characteristics	N (%)	Rate/1000 children*	Patient/Injury characteristics	N (%)
Gender			Nature of injury (one most severe)	
Male	1778 (65.9)	20.3	Cuts, bites or open wound	1378 (51.1)
Female	918 (34.1)	10.6	Bruise or superficial injury	383 (14.2)
Age groups (years)			Fracture	299 (11.1)
0–4	653 (24.2)	17.1	Sprain, strain or dislocation	243 (9.0)
5–9	866 (32.1)	17.5	Internal injury	124 (4.6)
10–14	680 (25.2)	12.0	Head injury/Concussion	83 (3.1)
15–17	497 (18.4)	16.6	Burns	67 (2.5)
0–17	2696 (100)	15.5	Other	115 (4.3)
Ethnicity/Caste**†**			Unknown	2 (0.1)
Janajati	1384 (51.3)	–	Not recorded	2 (0.1)
Brahmin/Chhetri	892 (33.1)	–	Activities at the time injury occurred	
Dalit	148 (5.5)	–	Leisure/Play	1889 (70.1)
Madhesi	146 (5.4)	–	Travelling (not to/from school/work)	296 (11.0)
Muslim	74 (2.7)	–	Work	202 (7.5)
Others/Unknown	52 (2.0)	–	Travelling (to/from school/work)	184 (6.8)
Place where injury occurred			Education	42 (1.6)
Home/Compound	1576 (58.5)	–	Organised sports	11 (0.4)
Highway/Road/Street	636 (23.6)	–	Other	52 (1.9)
School	233 (8.6)	–	Unknown	20 (0.7)
Recreational area	138 (5.1)	–	Disposition	
Workplace	76 (2.8)	–	Discharged	2317 (85.9)
Other	37 (1.4)	–	Admitted to hospital	164 (6.1)
Severity of injury**‡**			Transferred to another hospital	179 (6.6)
No apparent injury	125 (4.6)	–	Died	21 (0.8)
Minor	1645 (61.0)	–	Leave against medical advice	11 (0.4)
Moderate	813 (30.2)	–	Unknown	2 (0.1)
Severe	111 (4.1)	–	Not recorded	2 (0.1)
Not recorded	2 (0.1)	–	–	–

*Rates calculated using population estimates for Makwanpur from the 2011 census. Census data do not enable reporting of rates by ethnicity/caste.

†Ethnicity/Caste classification as per Nepal Health Management Information System.

‡Injury severity was classified as ‘minor’ (superficial injury such as bruises or cuts), ‘moderate’ (injures requiring skilled treatment) or ‘severe’ (injures requiring intensive management) depending on the level of requirement for skilled emergency care intervention.

The majority (95%) of injuries presenting to the emergency department were reported by parents to have occurred unintentionally. Injury data by unintentional and intentional mechanisms are shown in table 2.

**Table 2:** Distribution of injuries by age group, sex and mechanism of injury among children attending emergency of hospitals in Makwanpur district, Nepal, April 2019–March 2020

Age groups and sex	0–4 years	5–9 years	10–14 years	15–17 years	Male	Female	Total	
Intent and mechanisms	N (%)	N (%)	N (%)	N (%)	N (%)	N (%)	N (%)	Rate/1000 children*
Unintentional								
Fall	239 (36.9)	328 (38.5)	249 (38.4)	96 (23.2)	636 (37.4)	276 (32.2)	912 (35.6)	5.2
Animal or insect related	175 (27.0)	260 (30.5)	190 (29.3)	103 (24.9)	470 (27.6)	258 (30.1)	728 (28.4)	4.2
Road traffic injury	49 (7.6)	108 (12.7)	86 (13.3)	113 (27.4)	223 (13.1)	133 (15.5)	356 (13.9)	2.0
Injured by a blunt object	54 (8.3)	74 (8.7)	49 (7.6)	24 (5.8)	150 (8.8)	51 (5.9)	201 (7.9)	1.2
Stabbed, cut or pierced	20 (3.1)	56 (6.6)	49 (7.6)	51 (12.3)	127 (7.5)	49 (5.7)	176 (6.9)	1.0
Fire, burn or scald	42 (6.5)	10 (1.2)	9 (1.4)	4 (1.0)	27 (1.6)	38 (4.4)	65 (2.5)	0.4
Poisoning	33 (5.1)	6 (0.7)	5 (0.8)	8 (1.9)	26 (1.5)	26 (3.0)	52 (2.0)	0.3
Suffocation/Choking	24 (3.7)	5 (0.6)	2 (0.3)	5 (1.2)	20 (1.2)	16 (1.9)	36 (1.4)	0.2
Electrocution	2 (0.3)	0 (0.0)	3 (0.5)	7 (1.7)	10 (0.6)	2 (0.2)	12 (0.5)	0.1
Other	9 (9.5)	5 (0.6)	6 (1.0)	2 (0.5)	13 (0.8)	9 (1.0)	22 (0.9)	0.1
Total	647 (100)	852 (100)	648 (100)	413 (100)	1702 (100)	858 (100)	2560 (100)	14.7
Self-harm								
Poisoning	0 (0.0)	0 (0.0)	6 (46.2)	32 (69.6)	7 (41.2)	31 (70.5)	38 (62.3)	0.2
Hanging	0 (0.0)	0 (0.0)	3 (23.1)	9 (19.6)	4 (23.5)	8 (18.2)	12 (19.7)	0.1
Other	0 (0.0)	2 (100)	4 (30.8)	5 (10.9)	6 (35.3)	5 (11.4)	11 (18.0)	0.1
Total	0 (0.0)	2 (100)	13 (100)	46 (100)	17 (100)	44 (100)	61 (100)	0.3
Assault								
Bodily force (physical violence)	3 (50.0)	1 (8.3)	11 (57.9)	28 (73.7)	37 (62.7)	6 (37.5)	43 (57.3)	0.2
Injured by a blunt object	2 (33.3)	8 (66.7)	4 (21.1)	4 (10.5)	13 (22.0)	5 (31.3)	18 (24.0)	0.1
Other	1 (16.7)	3 (24.9)	4 (21.1)	6 (15.7)	9 (15.3)	5 (31.5)	14 (18.7)	0.1
Total	6 (100)	12 (100)	19 (100)	38 (100)	59 (100)	16 (100)	75 (100)	0.4

*Rates calculated using population estimates for Makwanpur from the 2011 census.

### Where did childhood injuries occur?

The most common location for childhood injury in this study was at home (n=1576, 58%) followed by the highway/road (n=636, 24%), together accounting for 82% of all injuries. While the majority of injuries sustained on the road/highway were due to road traffic (386/636, 61%), 160/636 children (25%) were injured by animal bites/stings and 90/636 (14%) by falls.

### What were children doing when they were injured?

The most common activity at the time of the injury occurrence was ‘leisure/play’ (70.1%), followed by ‘travelling’ (11%) and ‘work’ (7.5%). Most children injured during ‘work’ were aged 15–17 years, but it is noteworthy that 45% of children injured while working were aged 5–14 years.

### What do the data tell us about injury mechanisms in children?

Falls (35.6%), animal bites and stings (28.4%) and road traffic injuries (13.9%) accounted for nearly 75% of all child injuries presenting to emergency departments in this study. A large majority of animal-related injuries were from dog bites accounting for 555/728 (76%) of these injuries, followed by injuries from cats (5%), scorpions (5%) and snakes (4%). Animal bites mostly affected children aged 5–9 years (36% of all bite injuries). Some injury mechanisms were notably infrequent—drowning (0.3%); unintentional poisonings (2.0%); burns and scalds (2.0%).

### What types of injury did children sustain?

The majority of injuries (51.1%) presenting to emergency departments were cuts, bites and open wounds, followed by bruises/superficial injuries (14.2%), fractures (11.1%), sprains/dislocations (9.0%), head injury/concussion (3.1%) and burns (2.5%).

### Was there much self-harm and what kind?

Self-harm presentations accounted for 2.2% (n=61) of all child injury attendances in this study. Self-harm was the most common in females (72%) and mostly in children aged 15–17 years (75%). Over half (62.3%) of self-harm presentations were from poisonings/overdose followed by strangulation/hanging (20%). Intentional poisonings made up 43% of poisoning cases with pesticide and insecticide poisoning accounting for 38% of self-harm poisonings. There were no deaths from intentional poisonings.

### What about violence and assault?

There were 75 assault-related injury presentations representing 2.8% of all child injury attendances.

Assault was the most common in males (79%) and in those aged 15–17 years (50.7%). The most common causes of assault injuries were ‘bodily force’ (57.3%); being injured by a blunt object (24%) or being stabbed/cut (10.7%). These three injury mechanisms together accounted for 92% of assault injuries. Alcohol was identified as a factor in 9% of injuries ensuing from violence and assault but other drugs were not implicated in cases.

### Injury severity and disposition

In this study, 61% (n=1645) of injuries were recorded as ‘minor’ with ‘moderate’ severity injuries accounting for 30.2% (n=813) of injury presentations. Severe injuries accounted for 4.1% (n=111) of childhood injury presentations. Age and sex associations with injury severity are shown in table 3.

**Table 3:** Association between injury severity and (i) sex; (ii) age group among children attending emergency of hospitals in Makwanpur district, Nepal, April 2019–March 2020

	Minor/Moderate n (%)	Severe n (%)	Total n (%)	χ^2^ (p value)
Male	1716 (66%)	60 (54%)	1776 (100)	7.261 (0.007)
Female	867 (34%)	51 (46%)	918 (100)	
Total	2583 (100)	111 (100)	2694 (100)	
<15 years	2128 (82%)	69 (62%)	2197 (100)	28.93 (<0.001)
15–17 years	455 (18%)	42 (38%)	497 (100)	
Total	2583 (100)	111 (100)	2694 (100)	

The in-hospital mortality for injured children in this study was around 1% (21 children). Ten of the 21 deaths (48%) were due to self-harm hanging/strangulation. Of the children that died, nine were 15–17 years of age (43%); six were aged 10–14 years (29%) and five were in the 0–4 age group (24%). The mechanisms of injury leading to death in children aged 0–4 years were road traffic injuries, choking/suffocation and drowning.

## Discussion

This study illustrated the feasibility of establishing injury surveillance in a government and private hospital in Nepal, and provides the largest prospective dataset published in recent years and the only one to yield so much data on the paediatric population.

Most childhood injuries in this study were falls, animal bites and stings and road traffic injuries and predominantly affected male children under the age of 10 years. This resembles systematic review findings relating to injury burden in Nepal which, though in all age groups, found that the leading types of injury were falls, road traffic injuries and cuts.^10^ The Nepal injury literature has previously shown that road traffic injuries are common in school aged children,^23^ and that falls were the most common cause of non-fatal injury in children.^24^ In contrast to the published literature which reported the importance of snakebites in children,^25^ we found that dog bites were a more significant issue. Our data support a focus on these three mechanisms for childhood injury prevention.

Burn injuries are a leading cause of morbidity and mortality in Nepal^26^; however, relatively few children with minor to moderate burns attend health facilities, with most receiving care at home.^27^ The relatively low proportion of childhood burns and scalds in this study is similar to that reported previously in this region.^17^ This may be due to poor access and transport, as much of Makwanpur district is rural and travel times are long. Previous community surveys of burn care suggest low knowledge of burn first aid (including appropriate cooling) and common use of lotions and pastes (eg, tomato, herbs and cow dung).^27^ Implementation and evaluation of campaigns to improve first aid and appropriate attendance at hospital appear warranted.

Studies reporting self-poisoning indicate this is a relatively common presentation, predominantly in young adults.^10^ Our study indicates the issue occurs in young people too. The data confirm previous findings of organophosphate pesticides as the predominant agent used in self-harm poisonings, while in unintentional poisonings the agents were often kerosene or medications. In this study, 40% of all poisonings were reported to be intentional, however, establishing intentionality is challenging, and this finding is likely to be affected by social desirability bias.

**Figure 2:**
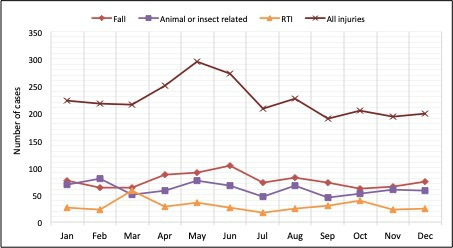
Seasonal variation of injuries identified by the injury surveillance system over a year among children attending emergency departments in Makwanpur district, Nepal, April 2019–March 2020. RTI, road traffic injury.

Previous studies reporting drownings in Nepal have shown children to be highly vulnerable^28^ and while the number of cases were relatively low, the data are in keeping with previous results.^17^ In neighbouring countries like Bangladesh and India, drowning is a leading cause of death, especially in children aged 1–4 years.^29 30^ Relatively low numbers of child drowning presentations to emergency departments in Nepal may reflect the fact that children may die at the scene of the event and not present to the emergency department. We did not observe any seasonal increase in drownings (or any other injury type) coinciding with the monsoon season between June and September (figure 2).

The study indicates groups of children vulnerable to specific injury types or to injuries occurring in specific locations; for example, dog bites in primary school aged children or road traffic injuries and self-poisoning in children aged 15–17 years. These data thereby present an opportunity for targeted prevention and policy interventions. Such targeted approaches have the potential to reduce injuries in children in this part of Nepal and more widely across the country. There are over 100 different ethnic groups in Nepal, and a number of different classification systems cluster ethnic groups variably. Janajati was the most common ethnic group in this sample, reflecting the predominance of the Tamang community in Makwanpur. Ethnic groups are associated with different castes which often determine parental occupation and family income. As injury occurrence is known to be associated with deprivation, further research on ethnic group distribution of injuries appears warranted, and our study has shown that it is feasible to collect complete ethnic group data. There is currently no national injury prevention strategy in Nepal, and although road traffic injuries are gaining attention, coordinated and funded action is lacking. This study therefore provides evidence to advocate for greater priority for this preventable burden of harm to children.

The main limitation of this study is that of selection bias because it will only have identified children with injuries presenting to the emergency departments. Data on children with injuries seeking care from hospitals outside the district, from community-based health services, local healers or receiving self-care will not have been captured. Our study will therefore have underestimated the true burden of injuries in the communities being studied, but is likely to have captured the majority of moderate and severe injuries. Regarding child injury deaths specifically, our anecdotal experience is that most patients who have died from trauma in this region are brought to the hospital as part of police investigations and for postmortem examination. These elements should be considered when interpreting the data and generalising the findings beyond the study setting. Strengths of the study are in its use of a structured programme of surveillance to collect all injury cases in the emergency departments reported over a period of 12 months. Comprehensive training and support for data collectors and regular feedback of collected data to clinical teams resulted in high levels of completeness and data quality.

While this study delivered a hospital-based injury surveillance system that was time limited, poststudy dissemination activities with both hospital management boards have indicated interest to establish sustainable in-house systems in the future. There are important lessons to be learnt from this study about undertaking injury surveillance. We would recommend prioritising engagement of local health partners, staff training on data collection, use of digital data tools and continuing support and feedback on the surveillance process. Data from injury surveillance can highlight opportunities for prevention and policy change.^31 32^ To support the development of evidence-based injury prevention and prehospital care in Nepal, the establishment of functioning hospital-based injury surveillance systems are an important approach.

## Conclusion

This study confirms that injuries affecting children in Nepal represent a significant issue. The data observed on injuries from falls, road traffic injuries and injuries related to animals suggest potential areas for injury prevention. This is the biggest prospective injury surveillance study in Nepal in recent years and supports the case for using injury surveillance to monitor child morbidity and mortality through improved data.

## Data Availability

Data are available on reasonable request. All data relevant to the study are included in the article or uploaded as supplementary information. All data relevant to the study are included in the article or are available on reasonable request.
